# *Plasmodium knowlesi*: the game changer for malaria eradication

**DOI:** 10.1186/s12936-022-04131-8

**Published:** 2022-05-03

**Authors:** Wenn-Chyau Lee, Fei Wen Cheong, Amirah Amir, Meng Yee Lai, Jia Hui Tan, Wei Kit Phang, Shahhaziq Shahari, Yee-Ling Lau

**Affiliations:** Department of Parasitology, Faculty of Medicine, Universiti Malaya, Kuala Lumpur, Malaysia

**Keywords:** *Plasmodium knowlesi*, Humans, Research, Malaria eradication

## Abstract

*Plasmodium knowlesi* is a zoonotic malaria parasite that has gained increasing medical interest over the past two decades. This zoonotic parasitic infection is prevalent in Southeast Asia and causes many cases with fulminant pathology. Despite several biogeographical restrictions that limit its distribution, knowlesi malaria cases have been reported in different parts of the world due to travelling and tourism activities. Here, breakthroughs and key information generated from recent (over the past five years, but not limited to) studies conducted on *P. knowlesi* were reviewed, and the knowledge gap in various research aspects that need to be filled was discussed. Besides, challenges and strategies required to control and eradicate human malaria with this emerging and potentially fatal zoonosis were described.

## Background

Malaria is one of the oldest infectious diseases. Despite the significant reduction of global malaria cases decade by decade, malaria remains a significant healthcare and economic burden to many countries, especially the developing nations around the tropical and subtropical regions [1, 2]. This disease is caused by different species of apicomplexan parasites belonging to the genus *Plasmodium*. Over the past few decades, healthcare professionals and general public were educated that malaria was caused by four species of human malaria parasites, namely *Plasmodium falciparum*, *Plasmodium vivax*, *Plasmodium malariae*, and *Plasmodium ovale* [3]. After entering the new Millennium, *Plasmodium knowlesi*, a simian malaria parasite, has been recognized as the fifth medically important *Plasmodium* [4–6]. In this Thematic Series honouring “The Primate Malarias” book [7], the stories and research breakthroughs of *P. knowlesi* are described and reviewed here.

**Fig. 1:**
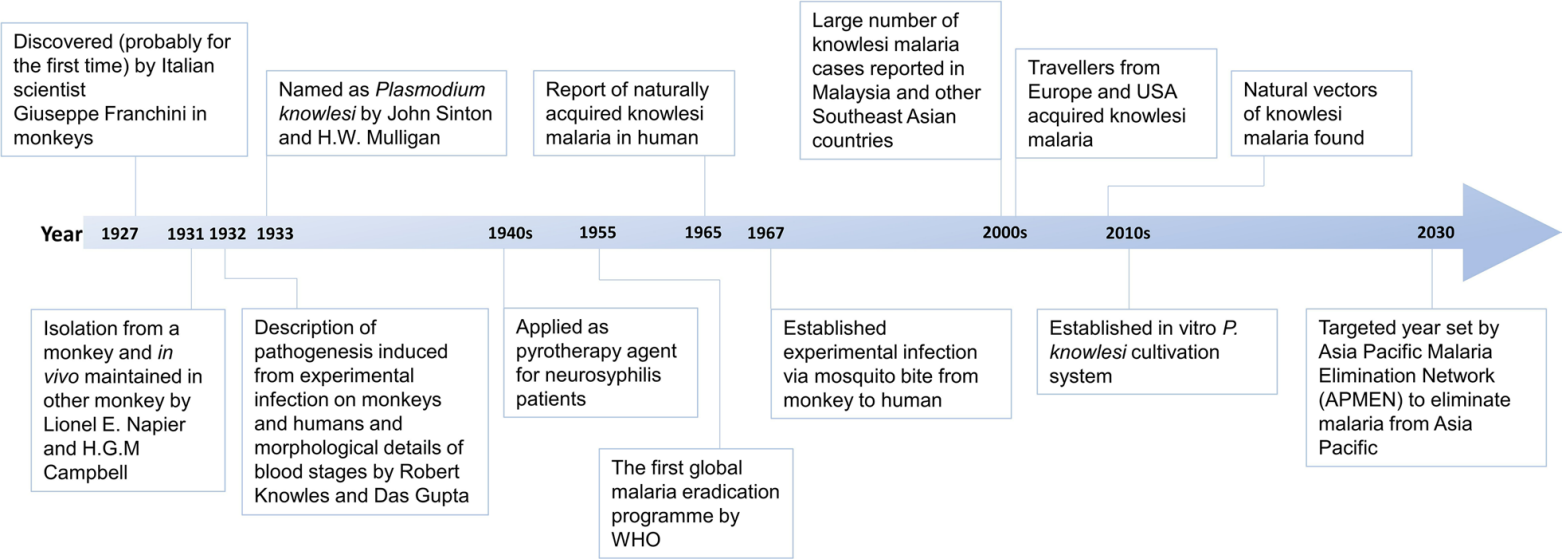
Schematic diagram showing some key events related to *P. knowlesi* in chronological order

The history of *P. knowlesi* discovery was relatively short (Fig. 1). The parasite was probably discovered by Giuseppe Franchini, an Italian scientist, while examining blood specimens of monkeys [8]. Subsequently, this parasite was successfully isolated and maintained in vivo using monkeys [9]. The detailed morphological description of the parasite staging, as well as the pathological profiles of different infected monkeys were described [10]. In addition, experimental infections on humans that gave rise to symptoms were described, providing the first recorded proof of pathobiological effects cast by this simian parasite to humans [10]. Not long after that, this parasite was named *P. knowlesi* [11]. Although fulminant disease experienced by human volunteers during the experimental *P. knowlesi* infection was reported, this simian malaria parasite was deemed ‘safe’. It was even used as a pyretic treatment agent for neurosyphilis patients [12]. Such treatment regime was stopped following reported deaths [13]. In 1965, a case of natural infection involving an American traveller returned from peninsular Malaysia was reported [14]. This was followed by another case suspected to be *P. knowlesi* infection acquired by a researcher from a trip to peninsular Malaysia [15]. Nevertheless, medical research attention given to *P. knowlesi* waned after the 1970s. The turning point for medical research attention on *P. knowlesi* happened when large clusters of *P. knowlesi* infections were detected in Malaysia [4, 5, 16], subsequently in almost all countries in Southeast Asia, with Timor-Leste as the only country that has yet to officially report any knowlesi malaria case to date (Table 1). The Southeast Asian region has become the epicentre of exporting knowlesi malaria to different parts of the world via frequent travelling and tourism activities (Table 1). The established knowlesi malaria transmission in Southeast Asia has challenged the malaria diagnosis approaches in this region. This zoonotic malaria has raised doubts whether malaria can be completely eliminated from the human populations in this region by year 2030, as set by the Asia Pacific Malaria Elimination Network (APMEN) [17].

**Table 1:** Southeast Asian countries with local knowlesi malaria cases and countries outside this region with reported cases

Name of countries/territories	Remarks	References
Cambodia	Two cases reported in Pailin province in 2010	[18]
Indonesia	An Australian working in forested area of South Kalimantan, Indonesian Borneo (2010); case clusters in North Sumatera, Indonesia (2015)	[19, 20]
Laos	A teenage boy living in a village surrounded by forest in Attapeu province, Laos (2016)	[21]
Malaysia	120 of the 208 malaria samples collected in Kapit division, Sarawak, Malaysian Borneo, from 2000 to 2002 were *P. knowlesi* infections; 77 of the 111 samples recruited from different states in peninsular Malaysia from 2005 to 2008 were *P. knowlesi* infections	[5, 22]
Myanmar	Mono-infections and mixed infections involving *P. knowlesi* were detected in Southern Myanmar in 2008	[23]
Singapore	Soldier acquired the infection from army training in forested area in 2007	[24]
Thailand	A Thai citizen from Bangkok travelled to hilly areas of Prachuap Khiri Khan province in Southern Thailand in 2000	[25]
The Philippines	Five cases detected in the Palawan province in 2006	[26]
Vietnam	*P. knowlesi* infections were detected in malaria samples collected from 2004 to 2006 in Ninh Thuan province	[27, 28]
China	A case from Yunnan province verified via PCR as knowlesi malaria, reported in 2006; and the first imported case (patient travelled to tropical forests of Malaysia) in 2014	[29, 30]
Finland	Patient travelled to peninsular Malaysia in 2007	[31]
France	Patient travelled to the west coast of Thailand in 2010	[32]
Germany	Patient travelled to Thailand from 25 December 2016 to 13 January 2017, visited a number of locations in Chiang Mai and Ranong provinces	[33]
India	Mono-infections and mixed infections of *P. knowlesi* were detected in the Nicobar and Andaman Islands of India, from 2004 to 2010	[34]
Italy	Patient acquired the infection from a trip to the Philippines in 2016	[35]
Japan	Patient acquired the infection from a trip to peninsular Malaysia in 2012	[36]
New Zealand	Patient acquired the infection from a trip to Malaysian Borneo in 2010	[37]
Poland	Patient acquired the infection from a trip to Sumatera, Indonesia in 2018	[38]
Scotland	Patient acquired the infection from a trip to Malaysian Borneo in 2012	[39]
Spain	Patient showed symptoms after returning from a six-month-trip to Indonesia, peninsular Malaysia, Thailand and Vietnam in 2009	[40]
Sri Lanka	Patient acquired the infection from forested area in peninsular Malaysia in 2016	[41]
Sweden	Patient acquired the infection from a trip to Malaysian Borneo in 2006	[42]
United States	The first reported naturally acquired *P. knowlesi* infection in human, acquired from a trip to peninsular Malaysia	[14]

Only the first reported natural and imported case reports from these sites were referred in this table (except for locations from a country that are geographically segregated, or closely related reports that require simultaneous referral). The non-Southeast Asian countries are presented in blue fonts, separated by a dotted line

### *Plasmodium knowlesi* and its natural hosts

The infections of *P. knowlesi* in humans are considered as accidents in the life cycle of this parasite. As humans are not the natural hosts, the pathobiology of *P. knowlesi* in human is different from that of its simian natural hosts. The pathogenesis and clinical pictures of knowlesi malaria has been described in detail elsewhere [43, 44]. Here, focus is given to the natural hosts of this parasites, the simians. To date, 13 species of non-human primate malaria parasites have been discovered, and seven of these are found in the macaque and leaf monkeys across Southeast Asia [45, 46]. *Plasmodium knowlesi* has been found in several species of these simian primates [7]. Ever since the discovery of persistent knowlesi malaria transmission across Southeast Asia, only a handful of surveys have been done to study the malaria parasites in their natural hosts (Table 2). Due to the difficulty in identifying the parasites up to the species level via microscopy, as well as the frequent occurrence of mixed infections in monkeys [47], only studies that used PCR were included in Table 2. It is unsurprising that the majority of studies were conducted in Malaysia as this is where the majority of knowlesi malaria cases are reported. A total of 3472 monkeys were screened in eight countries, with 75.5% (2623/3472) of the monkeys sampled being long-tailed macaques (*Macaca fascicularis*). The macaques caught from the Kapit division of Sarawak, Malaysia demonstrated the highest *P. knowlesi* infection rate, with an infection prevalence of 86.6% in the *M. fascicularis* population and 50% in the pig-tailed macaques (*M. nemestrina*) population [48]. Indonesia, Taiwan and Cambodia have yet to report any ‘*P. knowlesi*-positive’ monkeys [49, 50], whilst a study in Laos found one ‘*P. knowlesi*-positive’ long-tailed macaque from the 44 monkeys examined [49]. In the Philippines, *P. knowlesi* was detected in *M. fascicularis* from Puerta Princesa Subterranean River National Park, Palawan; but not in macaques from another wildlife centre (Palawan Wildlife Rescue and Research Center) in the same province [51].

**Table 2:** Studies of monkeys screened for simian malaria using PCR throughout Southeast Asia from 2008 to 2021

Countries/territories	Reference	Location	Sampling period	Monkey species sampled	Type of monkey	Total monkeys sampled	*P. knowlesi* positive samples	*P. knowlesi* infection prevalence (%)
Malaysia Borneo	Lee et al. [48]	Kapit Division, Sarawak	2004–2008	*M. fascicularis*	Wild	82	71	86.6
			2004–2008	*M. nemestrina*	Wild	26	13	50.0
	Muehlenbein et al. [55]	Sepilok Orangutan Rehabilitation Centre, Sabah	2010–2011	*M. fascicularis*	Wild	26	4	15.4
			2010–2011	*M. nemestrina*	Wild	15	2	13.3
Peninsular Malaysia	Vythilingam et al. [22]	Kuala Lipis Pahang	Not stated	*M. fascicularis*^*a*^	Wild	75	10	13.3
		Kuala Lumpur	Not stated	*M. fascicularis*^*a*^	Wild	29	0	0.0
		Selangor	Not stated	*M. fascicularis*^*a*^	Wild	41	0	0.0
	Ho et al. [56]	Selangor	Not stated	*M. fascicularis*	Wild	107	25^b^	23.3
	Khajeaian [57]	Peninsular Malaysia (Selangor, Negeri Sembilan, Pahang, Perak, Kelantan, Penang)^c^	2010 -2014	*M. fascicularis*	Wild	283	97	34.3
	Akter et al. [58]	Hulu Selangor, Selangor	2014	*M. fascicularis*	Wild	70	21	30.0
	Amir et al. [59]	Pahang	2016	*M. fascicularis*	Wild	34	9	26.5
			2016	*M. nemestrina*	Wild	5	0	0.0
		Perak	2016	*M. fascicularis*	Wild	26	1	3.8
		Johor	2016	*M. fascicularis*	Wild	38	1	2.6
Indonesia	Zhang et al. [49]	Southern Sumatra	2010	*M. fascicularis*	Wild	50	0	0.0
		Bintan Island (Island near Singapore)	2007	*M. fascicularis*	Wild	20	0	0.0
Singapore	Zhang et al. [49]	Singapore (unspecified)	2007	*M. fascicularis*	Wild	40	0	0.0
	Jeslyn et al. [52]	Military protected zone in Western Catchment Area	2007–2009	*M. fascicularis*	Wild	3	3	100.0
		Peridomestic from various parts of Singapore	2007–2009	*M. fascicularis*	Wild (Peri-domestic)	10	0	0.0
	Li [53]	Military protected zone in Western Catchment Area	2007–2011	*M. fascicularis*	Wild	93	45	48.4
		Peridomestic from various parts of Singapore	2007–2011	*M. fascicularis*	Wild (Peri-domestic)	65	0	0.0
	Li et al. [54]	Military protected zone in Western Catchment Area	2009–2017	*M. fascicularis*	Wild	379	145	38.3
		Peridomestic from various parts of Singapore	2008–2017	*M. fascicularis*	Wild (Peri-domestic)	660	0	0.0
The Philippines	Zhang et al. [49]	Zamboanga, Southern Philippines	2012	*M. fascicularis*	Wild	40	0	0.0
		Batangas, Northern Philippines	2012	*M. fascicularis*	Wild	28	0	0.0
	Gamalo et al. [51]	Puerto Princesa Subterranean River National Park, Palawan	2017	*M. fascicularis*	Wild	40	18	45.0
		Palawan Wildlife Rescue and Research Center, Palawan	2017	*M. fascicularis*	Captive	25	0	0.0
		National Wildlife and Research Centre, Diliman, Quezon City, Manila	2017	*M. fascicularis*	Captive	30	0	0.0
Taiwan	Huang et al. [50]	Chia-shan area Kao-hsiung City, sourthern Taiwan	2006–2008	*M. cyclopis*	Wild	51	0	0.0
		Southern Taiwan	2006–2008	*M. cyclopis*	Captive	235	0	0.0
Thailand	Putaporntip et al. [60] ^d^	Pattalung	2008–2009	*M. nemestrina*	Wild	13	0	0.0
			2008–2009	*M. arctoides*	Wild	4	0	0.0
		Pattani	2008–2009	*M. nemestrina*	Wild	1	0	0.0
			2008–2009	*M. fascicularis*	Wild	1	0	0.0
		Yala	2008–2009	*M. nemestrina*	Wild	62	0	0.0
			2008–2009	*M. fascicularis*	Wild	8	0	0.0
		Narathiwat	2008–2009	*M. nemestrina*	Wild	373	5	1.3
			2008–2009	*M. fascicularis*	Wild	186	1	0.5
			2008–2009	*Semnopithecus obscurus*^e^	Wild	7	1	14.3
	Fungfuang et al. [61]	Chacheongsao province	2017–2019	*M. fascicularis*	Captive	32	0	0.0
		Ranong province	2017–2019	*M. fascicularis*	Wild	4	0	0.0
		Prachuap Kiri Khan province	2017–2019	*M. arctoides*^e^	Wild	32	1	3.1
		Nakornatchasima province	2017–2019	*M. leonina*	Wild	25	0	0.0
Cambodia	Zhang et al. [49]	Vanny	2011	*M. fascicularis*	Wild	54	0	0.0
Laos	Zhang et al. [49]	Laos (unspecified)	2013	*M. fascicularis*	Wild	44	1	2.3

^a^Within these samples there is one *M. nemestrina* and one *Presbytis melolophus*. However, it is not stated where these two monkeys were obtained from

^b^Absolute value was not stated in the paper

^c^Unable to accurately discern the prevalence in the individual states

^d^A single round PCR reaction was done that amplified *Plasmodium* and *Hepatocystis*. Species were identified by cloning the PCR fragments and sequencing 10 positive clones per sample. Species specific PCR was not conducted and therefore, some species may have been missed due to stochastic effects

^e^Putative new host for *P. knowlesi*

Of note, captive and peri-domestic monkeys screened were negative for *P. knowlesi* (Table 2). This may be due to a lack of compatible vectors in the areas where these monkeys were kept [52–54]. This was clearly shown in a study conducted by Li et al. [54] that investigated wild *M. fascicularis* caught in a military protected area within the Western Catchment Area in Singapore and peri-domestic *M. fascicularis* caught in various locations throughout Singapore. *Plasmodium* infections were prevalent among the wild macaques whereas their peri-domestic counterparts were *Plasmodium*-free, suggesting that these peri-domestic macaques that are in close contact with humans, currently pose a low risk as the source of zoonotic malaria transmission in Singapore. However, this study also highlighted an increasing trend in the prevalence of *P. knowlesi* infection among the wild macaques caught in the Western Catchment Area from year 2009 to 2017. Notably, this study hypothesized that the reduction of macaque population in the area under study could lead to the higher mosquito biting frequency per macaque for the remaining macaques in this area. The attempts to control the monkey population in this area may be detrimental to the control of knowlesi malaria transmission as this could increase the prevalence of *P. knowlesi* infections in the remaining macaque population, resulting in greater risk of spillover infections to humans. Nevertheless, it is important to point out that most if not all the samples in this study were collected through the years and processed at about the same time. Whether the observed trend was due to increased transmission within the macaque population or a mere deterioration in the sample quality of the older samples remains unclear and deserves further investigations. Nevertheless, this report highlights the persistent presence of *P. knowlesi* reservoir in this highly developed nation.

As the natural transmission of knowlesi malaria from human to human via mosquito bite remains to be validated, the zoonotic transmission path is still regarded as the main route of knowlesi malaria acquisition in humans. In fact, recent studies have linked close macaque contact by humans with an increased risk of acquiring *P. knowlesi* infection [62, 63]. However, several locations with reported natural infections of knowlesi malaria in humans (such as certain parts of Myanmar, the Smith Island and Car Nicobar from the Andaman Archipelago) have no known macaques with the established status of ‘*P. knowlesi* natural hosts’ [23, 29, 34, 64]. This opens the possibility that there may be additional reservoir hosts for *P. knowlesi* other than the three established natural hosts, i.e. *M*. *fascicularis*, *M. nemestrina*, and the banded leaf monkeys (*Presbytis melalophos*) [7]. Moyes et al*.* suggested the Northern pig-tailed macaque (*M. leonina*) in Shan state of Myanmar as a potential host for *P. knowlesi* as it is closely related to *M. nemestrina* [64]. However, it should be noted that *P. knowlesi* has yet to be found in *M. leonina* (Table 2). Meanwhile, *P. knowlesi* was found in a stump-tailed macaque (*M. arctoides*) in Prachuap Kiri Khan province, Thailand, based on nested PCR method without further backing of evidence by the gold standard microscopy or other molecular tools like sequencing [61]. In another study, *P. knowlesi* was detected in a dusky leaf monkey (*Semnopithecus obscurus*), as confirmed by sequencing and phylogenetic analysis [60].

However, an issue remains with the Smith Island and Car Nicobar, as there are no known monkey populations that are native to these areas [64]. Other than the presence of *M*. *fascicularis* in the Port Blair Zoo on Smith Island [65], there are no reports of captive or introduced macaques in Car Nicobar. Thus, the natural infections of human knowlesi malaria in this region could have originated from any of the established natural host simians that are available on the islands but unreported, or a yet-to-be-identified simian reservoir. It is also possible that the human-to-human transmission has established in these areas. There does not seem to be any physiological barriers restricting *P. knowlesi* transmission via the human-vector-human route, as demonstrated in experimental infections [66]. Instead, the human-to-human transmission is likely to be hampered by ecological factors, such as the lack of suitable vectors in human dwellings, as the established vectors of *P. knowlesi* i.e. several members of *Anopheles leucosphyrus* group are known to be primarily forest-dwelling [67]. There may be yet-to-be identified knowlesi malaria vectors that can adapt to living and breeding at sites closer to human dwellings on these islands, which will facilitate human-to-human transmission. Succinctly, the picture of *P. knowlesi* transmission is far from complete, especially in certain locations.

Although the human-to-human transmission of knowlesi malaria via *Anopheles* has yet to be validated or disputed, it should be noted that there is abundant evidence demonstrating that *P. knowlesi* is primarily a zoonosis. Many phylogenetic and haplotype network analyses did not show unique clusters associated with human cases, strongly suggesting that most, if not all of the human cases are originated from macaques [48, 52]. Furthermore, different studies have also indicated that the transmission of *P. knowlesi* in the macaque population is much higher than in the human population, implying that the human *P. knowlesi* infections are mainly the spill-over infections from the macaque population via biting activities by anopheline mosquitoes that bite both humans and monkeys [48, 68]. Of note, the *P. knowlesi* parasites derived from *M. fascicularis* and *M. nemestrina* in Borneo had distinct microsatellite genotypes, and human cases were associated with either the *M. fascicularis*- or *M. nemestrina*-derived parasite subpopulations, indicating that the majority of the clinical knowlesi malaria cases in Borneo were of zoonotic nature [68]. However, a small number of human cases showed admixtures of the two parasite populations, suggestive of possible human-to-human transmission at a much smaller scale. Similarly, Grigg et al. [62] and Fornance et al. [63] found a number of *P. knowlesi* cases within household members of a known *P. knowlesi* case, suggesting the presence of peri-domestic transmission. Obviously, the human-to-human transmission of knowlesi malaria could not be ruled out. The feasibility of simian-independent knowlesi transmission among humans will influence the strategies required to control and eradicate malaria in the affected areas. Hence, the feasibility of natural knowlesi transmission from human to human has to be investigated further.

### Individual and environmental factors associated with *P. knowlesi* infection

An accurate identification of potential risk factors associated with the transmission of *P. knowlesi* infection plays a crucial role in disease intervention and prevention. Epidemiology studies in recent years have led to the identification of various factors that influence the disease occurrence, which can be classified as individual and environmental factors (Table 3).

**Table 3:** Risk and protective factors associated with *P. knowlesi* infection and exposure

Categories	Risk/protective	Factors	Source
Individual	Risk	Age	[62, 63, 69, 70, 75, 79]
		Male Race Direct contact with monkeys	
		Forest-related and/or agricultural work (farmer, oil palm plantation worker, and vegetation clearing)	
		Travel into the forests/ eco-tourism	
		Sleep outside the house	
		Stay overnight in forest or in workplace near forest	
		Previous malaria infection	
		Lack of usage of personal protection (bed net, repellent/ residual insecticide spray)	
	Protective	G6PD deficiency	[62, 70]
		Personal protection (bed net, repellent/ residual insecticide spray)	
		Lived in village	
Environmental	Risk	Areas with significant forest coverage (within 2 km radius)	[62, 80, 81]
		Rapid deforestation (within 2 km radius)	
		Oil palm plantation and fragmentation of oil palm plantations	
		Patches of dense forest/ fragmentation of forests	
		Presence of wild monkeys	
		Long grass around house	
		Open roof eaves/gaps in house walls	
	Protective	Altitude elevation	[62, 80]
		Rice paddy fields around house	

Individual factors such as age, gender, outdoor activity engagement, types of outdoor activities involved, and occupation have significant impact on an individual’s risk of acquiring knowlesi malaria. Adults or individuals above 15 years old have been shown to have a greater risk of disease exposure [62, 69, 70]. From the socioeconomic viewpoint, a lot of people in rural areas of many Southeast Asian countries have started their working life at relatively young age (mainly in agriculture, forest resource collecting, hunting and logging industry) to lift the economy burden of their families, which agrees well with the findings of these reports. Apart from that, knowlesi malaria patients with older age have been associated with higher parasitaemia and greater risk of developing severe knowlesi malaria [71–73]. Besides, gender is also a risk factor. Males made up to over 80% of the cases reported in various studies [63, 74–76]. This again, is associated with socioeconomic structure of the community in the affected areas, where most of the labour-intensive jobs in the forests and farms are participated by males. Moreover, the gender bias is also reflected in other social activities such as jungle trekking, relaxing or sleeping outside the house, outdoor gatherings at night, and direct contact with monkeys. Collectively, these contribute to the higher risk of acquiring knowlesi malaria for males. Nevertheless, protective measures such as application of bed nets, insecticides, and residual spraying of insecticides, as well as staying in well-developed village were reported to reduce the risk of contracting *P. knowlesi* infection. Interestingly, hereditary conditions have also been suggested as a protective factor against knowlesi malaria, similar to those of falciparum malaria in Africa [77] and vivax malaria in Pakistan [78].

Ecological variations, both natural and human-induced, are the direct drivers of *P. knowlesi* transmission, as demonstrated in Sabah, Malaysia; where the shrinking of primary forest coverage has been associated with the increasing cases of *P. knowlesi* infections [80]. Loss of habitats due to deforestation forces the monkey population to shift into remaining forest patches and human settlements, increasing the chance of close contacts between humans and monkeys. Therefore, the presence of monkeys in human settlements or areas with human activities has also been shown to be a risk factor. Agricultural practices, such as irrigated farming, pulpwood plantation, and fragmented oil palm plantation, are risk factors as well, since these opened lands are usually at the fringe of forests [63]. Usually, the workers [for plantation, logging, hunting and natural resource collecting (bird’s nest, rattan etc.) industries] have to spend long hours near or within the forests, even in the late evenings (biting hours of *Anopheline* mosquitoes). As a result, they are exposed to infective mosquito bites. At peri-domestic and household-level, long grass around the house, and open roof eaves or gaps in house walls are welcoming signs of mosquito invasion, hence the risk factors of knowlesi malaria exposure. Interestingly, having rice paddy fields around the house, as well as residing at areas with higher altitudes are associated with lower infection risk [62]. These are probably associated with the availability of the vectors in these places, where the change in ecological factors limits the distribution of the knowlesi malaria vectors. In addition, application of various technology and methods have been employed to evaluate the spatial and temporal factors involved in the dynamics of knowlesi malaria transmission [81–85]. Collectively, these efforts enable a more precise and accurate risk prediction and forecast, which is useful for subsequent urban planning in the affected areas.

### Methods for detection of *P. knowlesi* infection

With the addition of *P. knowlesi* to the list of medically important malaria parasites, the standard operating protocols of malaria diagnosis in knowlesi malaria-endemic areas have to be adjusted to enable accurate detection of all etiological agents of malaria. To date, microscopic examination remains as the gold standard for the diagnosis of malaria. Although limitations have been described, i.e. time consuming and low sensitivity [86], this diagnostic method allows identification of parasite species and quantification of the parasite density in malaria endemic area when performed by skilled microscopists [87, 88]. The reliance on microscopic examination was challenged by the morphological similarities of *P. knowlesi* parasites with other human malaria species [89]. Early erythrocytic stages (ring forms) of *P. knowlesi* resemble the ring forms of *P. falciparum*. At the late trophozoite stage, *P. knowlesi* may appear as band forms, which resemble those of *P. malariae.* Hence, misidentification of knowlesi malaria as infections by *P. falciparum* or *P. malariae* is common, especially in areas where the microscopists are not familiar with the parasites [32, 37, 38, 90, 91]. Besides, parasites with atypical amoeboid morphology were also found in patient’s blood smears [92]. Misidentification of *P. knowlesi* as *P. vivax* were reported previously [91, 93]. *P. knowlesi* patients were given treatment with primaquine, which is a radical cure to clear hypnozoites (dormant liver stage) of *P. vivax*. This resulted in unnecessary increase of treatment cost and potential health risks to individuals with glucose-6-phosphate dehydrogenase (G6PD) deficiency if G6PD testing was not performed prior to the treatment, which could lead to severe acute haemolytic anaemia [94]. Therefore, differential diagnosis is essential in distinguishing knowlesi malaria from malaria caused by other species of *Plasmodium*, by taking into considerations the parasite’s morphology and patient’s travel history prior to the infection.

Malaria rapid diagnostic test (RDT) is a convenient alternative for malaria diagnosis due to its ease of use, low cost and rapid yield of results. The tests are immunochromatographic lateral flow devices that provide qualitative results, which is especially valuable in resource-limited settings and mass screenings. Current malaria RDTs target three proteins, namely *P. falciparum* histidine-rich protein 2 (PfHRP2), plasmodial lactate dehydrogenase (pLDH) and plasmodial aldolase. Antibodies targeting these antigens are used for specific detection of *P. falciparum* or *P. vivax*. They are also used in combination with pan-malarial antibodies that target all *Plasmodium* species [95]. The performance of these RDTs in diagnosing *P. knowlesi* infection has been evaluated. Foster et al. found that OptiMAL-IT was the most sensitive RDT in detecting *P. knowlesi* antigen, with 71% sensitivity for fresh samples [96]. However, the test also yielded false positive results at the *P. falciparum* test line, suggesting that the *P. falciparum* lactate dehydyrogenase (LDH) monoclonal antibody used in this kit cross-reacts with the *P. knowlesi* LDH antigen, which is in agreement with findings from other studies [97–99]. A systematic review on different RDTs revealed that the overall performance of currently available RDTs in detecting *P. knowlesi* remained low [100]. Thus, there is a need to design a RDT that is sensitive and specific for *P. knowlesi* detection, without compromising its performance of detecting malaria caused by other species of *Plasmodium*. Of note, Krause and Goldring reported the potential of phosphoethanolamine-N-methyltransferase (PMT) as a biomarker candidate for RDT design due to its presence in erythrocytic stages of the *Plasmodium* parasites [101]. The protein also shares a relatively low similarity across the *Plasmodium* species orthologues. Hence, the production of a RDT with PMT monoclonal antibodies targeting species-specific epitopes is deemed possible.

Despite of its limited use in diagnosing acute infections, serological assay plays an important role in malaria disease surveillance, screening in blood donation centres, and identification of parasite exposure history [102]. There are several malaria ELISA kits that detect anti-*Plasmodium* antibodies. Most of the tests used *P. falciparum* and *P. vivax* recombinant antigens as antigenic targets*.* A commercial ELISA (EUROIMMUN EIA) which used recombinant antigens from all five medically important *Plasmodium* species was shown to exhibit high concordance rate to routine screening test (> 94%), with sensitivity and specificity of 85% and 95.2%, respectively [103]. Using the available genome sequences of *Plasmodium* parasites, Müller-Sienerth et al. expressed and evaluated a panel of recombinant proteins to be used as target antigens in ELISA [104]. The study discovered that *P. knowlesi* merozoite surface protein 10 (PkMSP10), 6-cysteine protein 12 (PkP12), and 6-cysteine protein 38 (PkP38) could be used as antigen panels in serological assays as they accurately determined the patient’s history of exposure to *P. knowlesi*.

Microscopy and RDT have a detection limit of 100 and 5–50 parasites/μL, respectively [105]. This leads to a major challenge for early diagnosis of *P. knowlesi* at low parasitaemia [73]. Besides, asymptomatic individuals are also difficult to be detected via microscopy and RDT. To mitigate such limitations, molecular diagnostic methods have emerged as an alternative tool for the detection of *Plasmodium* up to species level, with the ability of detecting much lower load of parasites in the samples. The polymerase chain reaction (PCR)-based diagnosis has gained popularity among the researchers. Such methods encompass nested PCR, real-time PCR and multiplex PCR. The PCR-based diagnoses yield much higher sensitivity than microscopy in the detection of *Plasmodium* spp. [106]. Hofmann et al. reported that most of the PCR-based diagnoses possess a common lowest detection limit of one parasites/µL [107]. Moreover, the PCR-based diagnoses are effective in detection of mixed infections, screening large number of samples within a short period of time, and studying drug resistance-related markers in the parasites. Ten years ago, Hindson et al. reported the use of a novel molecular technology known as the droplet digital polymerase chain reaction (ddPCR) to quantitate DNA [108]. Since then, various malaria researchers have explored the potential of using ddPCR as the better diagnostic approach. Recently, Mahendran et al. developed duplex ddPCR for *P. knowlesi* and *P. vivax* detection, which yielded superior sensitivity of detection, as compared with the established nested PCR method [109]. When compared with the quantitative PCR (qPCR), ddPCR assay can be performed without the need of generating a standard curve. The ability of this assay to detect *P. knowlesi plasmepsin gene* as low as 0.01 copies/µL further highlighted its potential in detection of low-density malaria cases [109]. The findings were in agreement with an earlier report using 150 clinical samples, where ddPCR was found to detect more *P. falciparum* infections than qPCR, and both methods diagnosed an equal number of *P. vivax* infections [110]. In addition, this earlier study also reported that ddPCR managed to identify more mixed infections than qPCR.

The PCR-based methods are not without limitations. The major drawbacks of PCR-based assays include the requirement of expensive machines, costly reagents, longer turnaround time and a well-equipped laboratory, which restrict its application for field diagnoses. Furthermore, it is not suitable to be used in countries with low resource settings and unstable power supply. As an alternative, isothermal methods have been pushed forward. Since year 2000, loop-mediated isothermal amplification (LAMP) has been widely used to assist malaria diagnosis in some areas, due to its highly sensitive and rapid performance, in addition to its requirement of cheaper equipment and resources [111]. With an incubation in a heating block at 65 °C, diagnostic results of LAMP can be obtained after ~ 45 min. LAMP method that can identify all four species of human malaria parasites were developed and reported in year 2007 [112]. Subsequently, Lau et al. developed a species-specific LAMP approach that covered the five medically important parasites (*P. falciparum*, *P. vivax*, *P. malariae*, *P. ovale*, and *P. knowlesi*), with a detection limit of one copy/µL for *P. vivax*, *P. falciparum*, and *P. malariae*; and ten copies/µL for *P. knowlesi* and *P. ovale* [113]. To facilitate the end point detection of LAMP, a variety of DNA intercalating dyes [113–115], fluorescent indicator dyes [116, 117] and pH indicators [118] can be included in the LAMP assay. Recently, Lai et al. has developed a SYBR green I LAMP assay for the detection of *P. knowlesi* by targeting the *18s rRNA* gene, where a positive reaction is indicated by green colour whereas the negative reaction is indicated by orange colour [119]. This new assay exhibited clinical sensitivity of 97.1% and clinical specificity of 100%. Besides, LAMP has the potential to be developed as a point-of-care (POC) diagnosis tool with the combination of lateral flow technology. The combination of LAMP and lateral flow dipstick (LAMP-LFD) is an innovative method to analyse various samples in the field setting. A positive sample will generate a signal at both control (C) and test (T) lines. Yongkiettrakul et al. developed a LAMP-LFD assay for simultaneous detection of *P. falciparum* and *P. vivax*, which showed a tenfold higher detection limit than nested PCR [120]. In 2018, Mallepaddi et al. designed a LAMP-LFD assay to detect human malaria parasites with a detection limit of 0.01 pg/μL for the five medically important *Plasmodium* species [121]. Another potential isothermal method to be employed for improved malaria diagnosis is the recombinase polymerase amplification (RPA). Compared to PCR-based assays and LAMP, RPA is more rapid (< 20 min) approach, and easier to perform as it requires lower temperature (37–42 °C) and amplifies DNA without the need of a thermo cycler. In fact, RPA has been established as a diagnostic tool for malaria [122, 123]. The combination of RPA and lateral flow dipstick (RPA-LFD) allows this technology to be integrated into POC testing [124–126].

In recent years, new generation of molecular diagnostic tools have been developed, one of these is the specific high-sensitivity enzymatic reporter unlocking (SHERLOCK) assay. This is a novel diagnostic approach, where CRISPR technology and RPA assay are combined. SHERLOCK is an ultrasensitive CRISPR-based method that allows the detection of infections from asymptomatic carriers [127, 128]. The Isothermal detection tools are more promising than the PCR-based methods. However, there are still rooms for improvement. To develop a reliable and user-friendly method, we should focus on the product’s innovation, simplicity of the approach and its cost-effectiveness. For instance, a method requiring minimal electricity supply is an advantage, particularly in many field settings. Sema et al. have reported a non-experimental nucleic acid amplification assay (NINA)-LAMP for the detection of *Plasmodium* species [129]. This technique requires only exothermic chemical reactions to generate heat energy needed by the LAMP assays. NINA-LAMP has the potential to be developed as a POC diagnostic tool. In short, the development of new diagnostic devices for resource-limited settings should follow the recommended guidelines by the World Health Organization (WHO), i.e., the ‘ASSURED’ criteria. ‘ASSURED’ stands for Affordable, Sensitive, Specific, User-friendly, Rapid and robust, Equipment-free and Deliverable to end users [123].

### Genetic structure and diversity of *P. knowlesi*

Information on the genetic diversity of a parasite species is crucial for the development of vaccines against malaria. High antigenic variation of proteins expressed by the parasites on the surface of infected cells may compromise the feasibility of generating protective immunity via vaccine and applying neutralizing antibody-mediated therapy on malaria patients [130, 131]. In fact, antigenic variation has been widely reported in *Plasmodium* spp. [132, 133]. The antigenic variation of *P. knowlesi* was first reported in 1965 [134]. Subsequent investigations revealed various putative variant antigen families throughout the genome [132, 135, 136]. The recent breakthroughs of *P. knowlesi* genetic studies are summarized in Table 4. With knowledge obtained from the genetic diversity studies, the characteristics of gene expression switching by *P. knowlesi* can be unravelled more systematically, providing an important foundation to understand the pathobiology of knowlesi malaria.

**Table 4:** Overview of recent studies on genetic diversity of *P. knowlesi*

Authors (year)	Gene/polymorphic marker	Gene function	Geographical origin	Host origin^a^	N^b^	Selection pressure	Population clustering or other findings
Individual gene studies							
Fong et al. (2016) [137]	Gamma protein region II (*PkγRII*), Duffy binding protein α region II	Erythrocyte invasion	Pen. Malaysia, Sabah, Sarawak	H	79	Purifying selection for PkγRII	2 distinct geographical clusters between Pen. Malaysia and Malaysian Borneo
Loh et al. (2016) [138]	Circumsporozoite (*csp*), *SSU rRNA*, merozoite surface protein 1 (*msp1*), cytochrome c oxidase subunit 1 (*cox1*)	Sporozoite development and hepatocyte invasion (*csp*), erythrocyte invasion (*msp1*)	Singapore, Thailand, Pen. Malaysia, Sarawak	H	24	–	*cox1* showed differentiation among *P. knowlesi* isolates based on geographical region
Yusof et al. (2016) [139]	A-type *18S SSU rRNA*, *cox1*	–	Pen. Malaysia, Sabah, Sarawak	H, Mf	210	–	Neutrality test indicated population expansion
Ahmed et al. (2016) [140]	Normocyte binding protein Xa (*NBPXa*)	Erythrocyte invasion	Pen. Malaysia, Sarawak, Sabah	H	56	Purifying selection	3 clusters: Type 1 and 2 found in Pen. Malaysia and Malaysian Borneo whereas Type 3 found only in Pen. Malaysia
Grigg et al. (2016) [141]	Dihydrofolate-reductase (*dhfr*)	Folate biosynthesis pathway and pyrimethamine resistance marker	Sabah	H	446	dN/dS ratio indicated potential purifying selection	1/3 of the infections were with *P. knowlesi dhfr* mutants. No mutations were found at 4 aa sites that deemed critical for pyrimethamine binding among all isolates, indicating no evidence of drug selective pressure in humans
Rawa et al. (2016) [142]	Rhoptry-associated protein 1 (*rap1*)	Parasitophorous vacuole formation following erythrocyte invasion	Pen. Malaysia	H	34	Purifying selection	2 clusters were identified
Yap et al. (2017) [143]	Merozoite surface protein 1 42 kDa region (*msp1*_*42*_)	Erythrocyte invasion	Pen. Malaysia, Sabah, Thailand, India	H, Macaque	39	Purifying selection within Malaysia isolates	–
Chua et al. (2017) [144]	Apical membrane antigen 1 (*ama1*)	Erythrocyte invasion	Sabah	H	36	Purifying selection	–
De Silva et al. (2017) [145]	Merozoite surface protein 1 (*msp3*)	Erythrocyte invasion	Pen. Malaysia	H	48	Purifying selection in Domain B	2 clusters were identified
Ahmed et al. (2018) [146]	*msp1*	Erythrocyte invasion	Pen. Malaysia, Sabah, Sarawak, Thailand	H, Macaque	76	Purifying selection	3 clusters were identified: Malaysian Borneo cluster, Thailand human and Thailand macaque cluster, and mixture of Pen. Malaysia and Thailand isolates cluster
Ahmed et al. (2018) [147]	Merozoite surface protein 1 paralog (*msp1p*)	–	Pen. Malaysia, Sarawak	H	40	Purifying selection	4 distinct geographical clusters within Malaysia
Ahmed et al. (2018) [148]	Thrombospondin-related adhesive protein (*trap*)	Sporozoite motility to mosquito’s salivary gland and invasion to host hepatocytes	Pen. Malaysia, Malaysian Borneo	H	40	Positive selection/balancing selection	–
Ahmed et al. (2018) [149]	*P. knowlesi* 6-cysteine protein (*pk41*)	Surface antigen	Pen. Malaysia, Sarawak	H	39	Purifying selection	3 clusters: 2 clusters of Sarawak human isolates and third cluster consisted of lab isolates
Yap et al. (2018) [150]	*msp1*_*42*_	Erythrocyte invasion	Pen. Malaysia, Sabah, Sarawak	H	83	Purifying selection, Neutrality test indicated balancing selection in Malaysian Borneo isolates but not in Pen. Malaysia	2 distinct geographical clusters between Pen. Malaysia and Malaysian Borneo
Fong et al. (2019) [151]	Erythrocyte-binding protein region 2 (*pkβII*)	Erythrocyte invasion	Pen. Malaysia, Malaysian Borneo	H	65	Purifying selection	2 distinct geographical clusters between Pen. Malaysia and Malaysian Borneo
Ahmed et al. (2019) [152]	Merozoite surface protein 4 (*msp4*)	Surface antigen	Pen. Malaysia, Sarawak, the Philippines	H	36	Purifying selection in Exon II	2 distinct geographical clusters between Pen. Malaysia and Malaysian Borneo
Ahmed and Quan (2019) [153]	Merozoite surface protein 7D (*msp7D*)	Erythrocyte invasion (putative function)	Pen. Malaysia, Sarawak	H	37	Positive selection in central region but purifying selection found in 5’ and 3’ regions	–
Ahmed et al. (2019) [154]	Merozoite surface protein 8 (*msp8*)	Erythrocyte invasion (putative function)	Pen. Malaysia, Sarawak	H	43	Purifying selection	2 distinct geographical clusters between Pen. Malaysia and Malaysian Borneo
Chong et al. (2020) [155]	*csp*	Sporozoite development and hepatocyte invasion	Pen. Malaysia, Sarawak, Sabah, Singapore	H	212	Purifying selection	–
Ng et al. (2021) [156]	*ama1*	Erythrocyte invasion	Pen. Malaysia, Sarawak	H	41	Purifying selection	2 clusters distinguished between Pen. Malaysia and Sarawak
Microsatellite genotyping, whole-genome sequence analyses and other polymorphic marker studies							
Lee et al. (2011) [48]	Mitochondrial (mt) genome, *csp*	Sporozoite development and hepatocyte invasion (*csp*)	Sarawak	H, Mf, Mn	82 csp, 54 mt genome	–	Mitochondrial genome analyses suggested that *P. knowlesi* underwent population expansion approximately 30,000–40,000 years ago and possibility of increased parasite admixture between macaque troops
Divis et al. (2015) [68]	Microsatellites (10 loci)	–	Pen. Malaysia, Sabah, Sarawak	H, Mf, Mn	599	–	2 clusters associated with either Mn or Mf
Assefa et al. (2015) [157]	Whole-genome	–	Sarawak	H	53	16/2381 (0.67%) genes showed signs of balancing selection with highest Tajima’s D value in *csp*	3 clusters: 2 clusters of Sarawak human isolates and third cluster consisted of lab isolates Evidence of long-term population expansion
Pinheiro et al. (2015) [158]	Whole-genome	–	Sarawak	H	7	–	2801/4623 genes (60.8%) are dimorphic (2 clusters)
Divis et al. (2017) [159]	Microsatellites (10 loci)	–	Pen. Malaysia, Sabah, Sarawak	H, Mf, Mn	182	–	3 clusters: 2 Malaysian Borneo cluster associated with either Mn or Mf and 1 Pen. Malaysia cluster
Benavente et al. (2017) [160]	Whole-genome (nuclear, mitochondria, and apicoplast genomes)	–	Pen. Malaysia, Sarawak	H, Mf, Mn	60 (nuclear genome), 114 (mt and apicoplast genomes)	–	3 clusters: 2 Malaysian Borneo cluster associated with either Mn or Mf and 1 Pen. Malaysia cluster Evidence of genomic regions with shared polymorphisms between 2 Malaysian Borneo subpopulation clusters
Divis et al. (2018) [161]	Whole-genome (nuclear, mitochondria, and apicoplast genomes)	–	Pen. Malaysia, Sarawak	H, Mf, Mn	80 (nuclear genome), 129 (mt genome), 65 (apicoplast genome)	–	High heterogeneity in the level of intercluster divergence was distributed across the genome, with long contiguous chromosomal blocks having high or low divergence
Benavente et al. (2019) [162]	Whole-genome	–	Pen. Malaysia, Sabah, Sarawak	H, Mf, Mn	103	–	*NBPXb* gene showed genetics exchanges between some Mn- and Mf-associated isolates DBPβ and *NBPXa* presented genetic exchange events with Mn-Pk into the Peninsular subpopulation
Saleh Huddin et al. (2019) [163]	Microsatellites (7 loci)	–	Pen. Malaysia	H, Mf	173	–	No significant genetic differentiation was seen between human and long-tailed macaque in Pen. Malaysia
Hocking et al. (2020) [164]	Whole-genome	–	Pen. Malaysia	H	28	215/4742 (4.53%) genes showed signs of balancing selection	3 subclusters were observed within Pen. Malaysia isolates
Divis et al. (2020) [165]	Bi-allelic SNP	–	Sabah, Sarawak	H, Mf, Mn	1492		2 clusters associated with either Mn or Mf.Cluster associated with Mf was the predominant (70%) infections.Majority of the recent cases were found to be grouped in Mf-associated cluster

*Pen. Malaysia* Peninsular Malaysia, *H* Human, *Mf* *M. fascicularis*, *Mn* *M. nemestrina*, *NBPXa* Normocyte Binding Protein Xa, *NBPXb* Normocyte Binding Protein Xb, *DBPβ* Duffy-binding Protein Beta, *aa* amino acid

^a^Host origin: most studies included genetic sequences of lab-maintained isolates in the analyses, hence, lab isolates were not specifically mentioned

^b^Number of sequences included in the analyses

To date, most of the *P. knowlesi* protein-coding genes, particularly those related to erythrocyte invasion, are experiencing purifying (negative) selection [137, 140, 147, 151, 166–168] that leads to the selective removal of deleterious alleles or less well-adapted variants, thus increases the frequency of the best-adapted beneficial variants within the parasite population [169]. Purifying selection may be an implication of functional constraints, in which the encoded proteins are prevented from losing their native functions in the course of evolution. Such selection has been proposed to be driven by the long-term population expansion of *P. knowlesi*, which could have been mitigated by the parasite population growth and adaptation to the mosquito vectors [48, 157]. However, some of these genes exhibit different selection pressures at different parts of the genes in different geographical locations [143, 145, 150, 153, 164]. In contrast to purifying selection, positive selection encourages the spread of genes that are advantageous to survivorship under sub-optimal conditions. Genes under positive selection include those encoded for proteins that are exposed to the immune system of the hosts, such as the variant surface antigens. The variation contributes to immune-escape mechanisms by the parasites. In *P. knowlesi*, only a few genes have been found to be under positive selection [148, 157, 164]. The *P. knowlesi* thrombospondin-related adhesive protein (TRAP) gene is one of such genes, which is similar to that of *P. falciparum* and *P. vivax* [148, 164, 170].

Through intensive studies of *P. knowlesi* genetic structure, we can understand the lineages of the parasites better. Phylogenetic studies allow us to understand the evolutionary progress of a species and its evolutionary relationship with other related species. For example, *P. knowlesi* shares a more recent common ancestor with *Plasmodium coatneyi*, another simian malaria parasite [55]. Dimorphisms, especially in association with geographical origins (between Peninsular Malaysia and Malaysian Borneo) were observed in many gene candidates [137, 150–152, 154, 156]. Following the application of multilocus microsatellite typing and whole-genome sequence analyses, three divergent subpopulations of *P. knowlesi* were unravelled in Malaysia [157, 159]. Microsatellite genotyping that targets multiple loci across the genome allows comprehensive screening of the whole genome at high resolution and identify loci that are under selection [161]. Studies based on whole-genome analysis revealed the existence of genomic mosaicism among the *P. knowlesi* subpopulations, indicative of chromosomal-segment exchanges events between two distinct Malaysian Borneo subpopulations associated with either *M. fascicularis* or *M. nemestrina* [160, 164]. On the other hand, another study reported that several fragments of genotype in the peninsular Malaysia subpopulation were similar to the *M. nemestrina*-associated Malaysian Borneo subpopulation [162]. Genetic studies enable large volume of complex information to be generated rapidly. Advances of technology in this field will drive this research niche even further. Efforts should be invested to ensure that the snapshots of information garnered from various studies are put together seamlessly to provide a concise and comprehensive picture.

### *Plasmodium knowlesi *in vitro culture adaptation and its research applications

The biological studies of a pathogen benefit tremendously from the establishment of a continuous cultivation system for the pathogen. This is clearly demonstrated in malaria research, where a greater depth of knowledge has been obtained from the large volume of in vitro studies conducted on the culturable *P. falciparum*, as compared to other human malaria parasites that have yet to be adapted successfully into the in vitro culture condition. Likewise, the rapid advancement in *P. knowlesi* research relies on the establishment of a reliable in vitro cultivation system for this species. Interestingly, the method used for *P. knowlesi* cultivation is largely based on the cultivation method established for *P. falciparum* in the 1970s [171]. The parasites are cultured in RPMI 1640 medium supplemented with serum, Albumax II (a serum substitute) or a combination of serum and Albumax II [172–174]. Earlier studies used rhesus serum to initiate *P. knowlesi* culture [172, 175], which was then adapted to human serum. However, subsequent studies have shown that *P. knowlesi* can adapt to the in vitro conditions directly using human serum and Albumax II [173], and even with Albumax II alone [174, 176], without the need of rhesus serum for initiation and adaptation. The parasites are maintained at a culture haematocrit level of 2–5% and low oxygen condition (2–5% O_2_, 5% CO_2_). Large amounts of parasites can be obtained consistently, which is important for high throughput studies. Rhesus or long-tailed macaque red blood cells (RBC) are used, where most studies require an adaptation period of about 3 weeks [172, 176]. Although *P. knowlesi* infects human RBC naturally, culturing the parasites in human RBC requires an adaptation process that takes around 5–8 months [173, 174, 177]. Two methods have been used to adapt *P. knowlesi* to human RBC invasion. The first method uses a mixture of human and macaque RBC (1:4 or 1:9 ratio of macaque RBC to human RBC) [173, 177]. The smaller fraction of macaque RBC allows the parasite population to be maintained, whilst the larger fraction of human RBC provides a selection pressure for the parasites to adapt to human RBC invasion. The second method explores the usage of human reticulocyte-enriched RBC [177]. In humans, *P. knowlesi* typically invades younger RBCs. Hence, *P. knowlesi* can be cultured with human reticulocyte-rich (~ 12%) packed RBC. The parasites are able to adapt to older RBC after a period of cultivation. This trick was utilized by a study group to adapt *P. knowlesi* parasites to human RBC without the need of macaque RBC. They initiated the culture using human RBC enriched with 16% reticulocytes, followed by gradual reduction of the reticulocyte fraction, over the course of five months [174].

The RPMI 1640-based in vitro cultivation method supports the growth of *P. falciparum* and *P. knowlesi*. The factors that enable *P. knowlesi* to adapt to human RBC in vitro using this cultivation protocol remain to be investigated and deciphered. Of note, the normocyte binding protein Xa (*NBPXa*) gene has been shown to be essential for human RBC invasion, but not for macaque RBC invasion [178]. In addition, Dankwa et al. discovered that the human-adapted *P. knowlesi* line created in their study contained a Duffy binding protein α (*DBPα*) gene duplication and a Duffy binding protein γ (*DBP*γ) gene deletion [174]. Hence, it was hypothesized that the adaptive ability of the parasites to invade human RBC in vitro might be attributed to the duplication of the *DBPα* gene. Meanwhile, a separate human-adapted *P. knowlesi* line demonstrated a V943L substitution in DBPα, which could be responsible for the adaptation to human RBC [173]. Thus, it seems likely that DBPα plays an important role in the adaptation of *P. knowlesi* to human RBC. Nevertheless, the role of the accompanying deletion of the *DBPγ* gene in such invasion adaptation is not known and deserves to be investigated as well, to completely decipher the invasion plasticity of *P. knowlesi* with RBC derived from different hosts. The successful adaptation of *P. knowlesi* to long term in vitro culture using human RBC has been a key turning point for knowlesi malaria research as it eliminates the need of macaque monkeys and macaque blood [173].

With the established culture system, the efficacy of anti-malarials, new therapeutic compounds or inhibitory antibodies have been assessed using growth inhibition assays (GIA). These assays can be performed with microscopic examination [179], radiolabelling of parasite DNA with [3H] Hypoxanthine [180], enzymatic-based evaluation by measuring activity level of *Plasmodium* lactate dehydrogenase enzyme [181], and flow cytometry to quantitate IRBC using DNA fluorescent dye [182]. Basically, studies that were previously conducted on *P. falciparum* are now applicable to *P. knowlesi*, with some adaptations and usage of newer, high throughput methods [183]. The in vitro susceptibility of *P. knowlesi* to various anti-malarials and novel therapeutic agent candidates have been evaluated using high throughput drug screening, thanks to the established in vitro culture system. Through these assessments, *P. knowlesi* has been revealed to demonstrate distinct drug susceptibility profiles, as compared to *P. falciparum*. For example, *P. knowlesi* has significantly lower susceptibility to a few sodium channel ATP4 inhibitors (a promising new anti-malarial target) [184–186]. Interestingly, other human *Plasmodium* species (*P. vivax, P. ovale* and *P. malariae*) demonstrated drug susceptibility profiles that were closer to that of *P. knowlesi* than *P. falciparum* [187]. With the establishment of such assessment platform, various *P. knowlesi* strains can be recruited for future evaluation of anti-malarials. Besides, attempts to in vitro induce drug resistance in *P. knowlesi* cultures can be done to investigate the development of drug resistance in this parasite, and predict gene candidates that drive the parasite towards drug resistance.

With the in vitro culture system, the effects of antibodies raised against a potential target can be evaluated, as part of the vaccine candidate screening. Indeed, the antibodies raised against both *P. knowlesi* Duffy binding protein α (PkDBPα) and apical membrane antigen 1 (PkAMA1) significantly inhibited parasite growth in a concentration-dependent manner [188], supporting the two candidates as potential vaccine candidates. In addition, the process of elucidating key ligands and receptors for various parasite-host interactions can be accelerated with a reliable in vitro cultivation system. Undeniably, this contributes to the better understanding of knowlesi malaria pathobiology.

Most of the adapted in vitro parasite cultures do not produce sexual-stage (gametocyte), possibly due to the loss of gametocytogenesis ability in the prolonged in vitro passage under well-regulated, optimal culture conditions [173, 189]. However, a *P. knowlesi* line maintained with macaque RBC was demonstrated to retain its ability to form gametocytes that gave rise to successful mosquito infection, as confirmed with the recovery of oocysts and sporozoites, albeit with inconsistency [176]. Nevertheless, with the possibility of generating infective gametocytes in vitro, investigations on the sporogonic cycle, characterization of gametocyte-specific genes/antigens, evaluation of transmission-blocking vaccine candidates, as well as gametocytocidal drugs against *P. knowlesi* are deemed feasible.

*Plasmodium knowlesi* is unique in that both in vitro and in vivo systems are available for research. Interestingly, the *P. knowlesi* maintained in in vitro cultures were shown to be capable of readapting to the in vivo conditions via a single blood passage into a macaque [172]. This allows *P. knowlesi* clones to be selected in vitro, subsequently used to infect a macaque (in vivo) to study the host-parasite interactions such as strain-specific virulence and factors that drive host tolerance upon infection. Notably, the in vitro-adapted *P. knowlesi* demonstrated clear difference in gene expression profile from the ex vivo parasites (i.e., cultured for only one cycle after withdrawal from a host in vivo), where the *SICAvar* genes were found to be downregulated in the in vitro cultures [190]. *SICAvar* is crucial for antigenic switching of *P. knowlesi*, possibly for the purpose of evading the host’s immune responses. The differences between in vitro and in vivo parasites have been demonstrated in *P. falciparum*, with differences being reported in gene expression and regulation, drug susceptibility, and cytoadherence characteristics [191, 192]. The availability of a research platform that allows relatively easy shuffling of a parasite between in vitro and in vivo systems allows more studies related to gene expression switching to be conducted. This is an obvious advantage of *P. knowlesi* research platform that is not available with *P. falciparum* and other human malaria parasites*.*

The human RBC-adapted *P. knowlesi* strain can be genetically modified with a conventional single or double crossover homologous recombination [173]. Remarkably, *P. knowlesi* demonstrated 1000-fold higher transfection efficiency than *P. falciparum*. Due to its shorter erythrocytic cycle, adequate quantity of transgenic parasite lines can be obtained within a week. The ability of the parasites to be cloned by limiting dilution allows transfection studies to be performed without the need of laboratory macaques, making functional studies on *P. knowlesi* cheaper, more ethical and more accessible to laboratories without monkey facilities. With that, targeted genes can be evaluated to decipher their roles in parasite multiplication/growth, and the specific interactions of ligands with the host receptors can also be investigated. For instance, disruption of *pkNBPXa* has led to impaired merozoite invasion into human RBC but not the macaque RBC, indicating *NBPXa* as a key mediator for human RBC invasion by *P. knowlesi* [178].

The CRISPR-Cas9 genome editing of *P. knowlesi* has been established [193], which will definitely drive the rapid and effective creation of transgenic parasite lines with precise gene editing, knock-out, or addition of tags to facilitate the downstream functional analysis of the parasite gene candidates. Moreover, this method can be used in combination with the conditional knockout system to generate parasite lines that are stable for inducible gene deletions to study the essential genes. In fact, *P. knowlesi* cysteine rich protective antigen (*PkCyRPA*) and RH5-interacting protein (*PkRIPR*) were demonstrated to be essential for the parasite’s survival and RBC invasion via the CRISPR-Cas9 system in combination with dimerisable Cre-recombinase (DiCre) system [194]. This breakthrough provides an important model system not only for *P. knowlesi,* but also other closely related species that lack a continuous in vitro culture system, such as *P. vivax*. Various studies on *P. vivax* have been performed using the orthologue replacement (OR) approach by creating chimeric *P. knowlesi* lines carrying the *P. vivax* ortholog genes to evaluate the drug resistance genes and vaccine candidates. For example, several *P. vivax* drug resistance markers including multidrug resistance protein 1 (*Pvmdr1*), dihydrofolate reductase (*Pvdhfr*), dihydropteroate synthase (*Pvdhps*) were expressed in *P. knowlesi* model system and their role in antimalarial resistance were assessed [195]. In addition, *P. vivax* Duffy binding protein (*PvDBP*) has been evaluated by using a stable *P. knowlesi* PvDBP^OR^ line, and the findings are favorable to support *PvDBP* as a leading *P. vivax* blood stage vaccine candidate [196]. Using the similar approach, other new vaccine candidate such as *P*. *vivax* 6-cysteine protein P12 (*Pv12*) and *P*. *vivax* Asparagine-rich Protein (*PvARP*) have been identified [197]. However, it is important to note that *P. knowlesi* does not share many of the morphological characteristics of *P. vivax* and does not form hypnozoites. The ability of forming hypnozoites is possessed by another simian malaria parasite, *Plasmodium cynomolgi,* whose long-term in vitro cultivation has been established recently [198]. Despites some limitations, the research potential and opportunity brought by the established *P. knowlesi* cultivation system is undeniably huge.

### Challenges to control and eliminate malaria with the emergence of knowlesi malaria

The ultimate goal of battling an infectious disease is the complete eradication of the infection from human population. Unfortunately, smallpox remains the only eradicated human infection in our history [199]. Ironically, malaria elimination program preceded smallpox elimination campaign by many years [200]. The malaria eradication program is a complex challenge involving various issues such as anti-malarial resistance development in the parasites [201], development of insecticide resistance among the anopheline vectors [202], political instability in a number of malaria endemic nations [203], and funding issues [204]. As mentioned earlier, malaria is caused by different species of *Plasmodium*. Each species has unique features such as the parasite carriage duration within the host, ability to form hypnozoites, infectivity to different species of *Anopheles*, and availability of reservoir hosts in the natural surroundings. In addition, different species of vectors have different characteristics, including different breeding ground requirements. Each of these features possesses different obstacles to the malaria eradication programme [205], reflecting the fact that the malaria eradication program should not be implemented with a “one-size-fits-all” mentality for all species of malaria parasites.

**Fig. 2:**
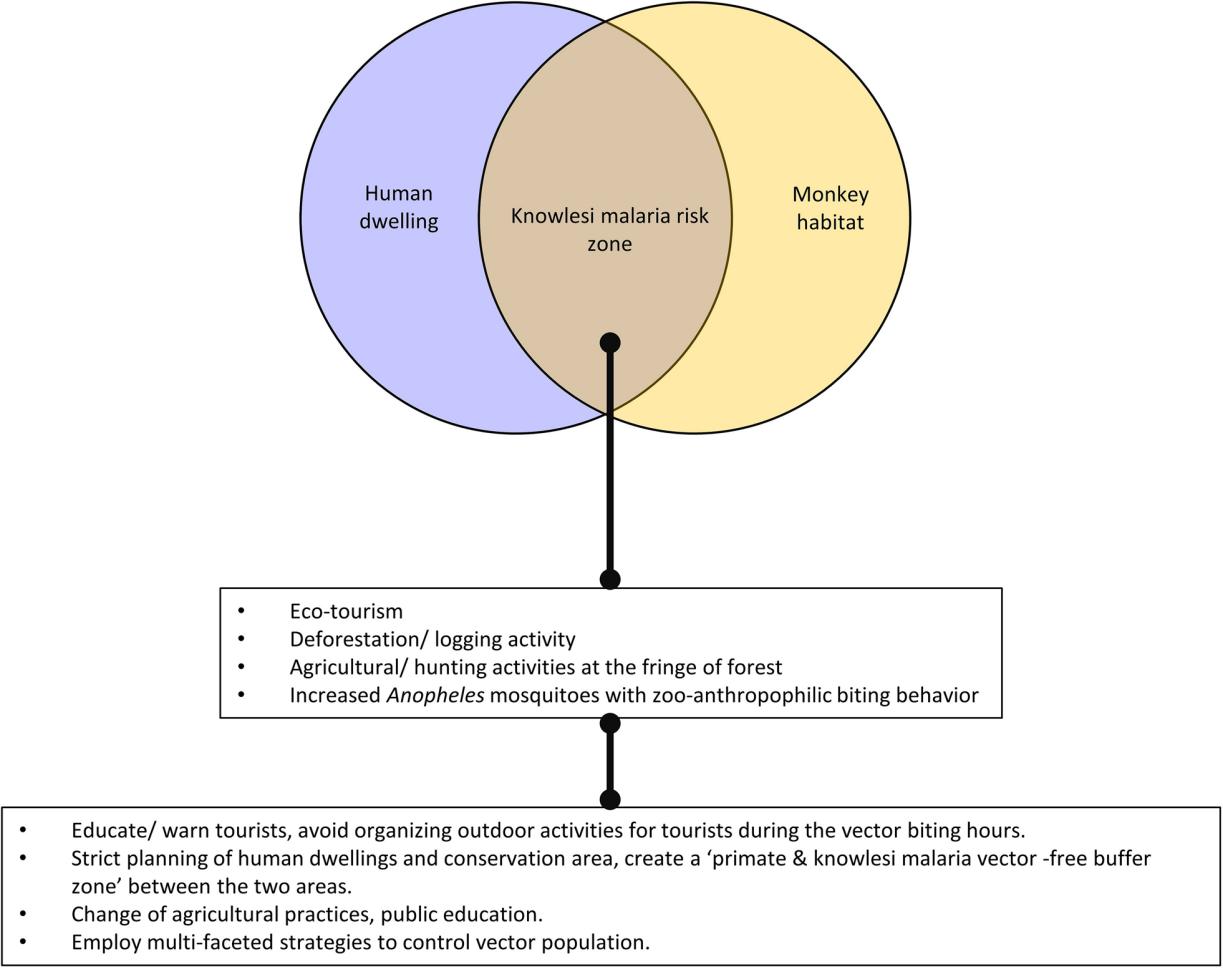
The dynamics of human dwelling and monkey habitat in transmission of knowlesi malaria, and possible strategies to break the circuit of transmission

The loop of knowlesi malaria transmission in humans involves several factors, i.e., the humans as accidental hosts, the natural host monkeys, along with the *Anopheles* that possesses zoo-anthropophilic feeding behaviour that can support the development of *P. knowlesi* salivary gland sporozoites. Various human activities such as logging industry, harvesting of jungle resources, subsistence cropping, expansion of housing development to the fringe of forests and eco-tourism at forested areas have brought humans closer to the natural hosts (monkeys) and vectors (*Anopheles*), completing the circuit of knowlesi malaria transmission in humans. Hence, the risk of knowlesi malaria transmission depends on the degree of overlap between the monkey habitats and areas with human activities (Fig. 2). The higher overlapping of areas used by humans and monkeys increases the risk of human knowlesi malaria transmission. Theoretically, measures that break this circuit of transmission will successfully halt the occurrence of knowlesi malaria in humans.

Obviously, the simian reservoirs cannot be culled just to get rid of *P. knowlesi* infection. Hence, the strategies to control and prevent transmission of knowlesi malaria have to be diverted to either the vectors or humans. For vector control, identifying all the vectors of knowlesi malaria is of utmost importance. To date, several species of mosquitoes from the *An. leucosphyrus* group have been incriminated as the vectors of knowlesi malaria [22, 206–211]. Application of insecticides may be of limited value because humans may be bitten by the *P. knowlesi*-infected *Anopheles* when engaging in various outdoor activities in forested areas, farms and plantations, since the vectors of knowlesi malaria have been shown to bite indoor and outdoor [212, 213]. Besides, mass-scale insecticide spraying at forested areas will threaten other non-target wildlife in these areas [214, 215]. Worse still, the natural predators of mosquitoes may be susceptible to insecticides, which may backfire the vector control effort and negatively affect the biodiversity of the targeted areas. Thus, a different vector control strategy is needed against knowlesi malaria transmission. Firstly, the approach of vector biocontrol using various natural predators of mosquitoes can be explored. For example, the feasibility of using larvivorous fish, nymphs of several commonly found odonate species, copepods, entomopathogenic fungi, and larvae of *Toxorhynchites* mosquitoes as the biocontrol agents against knowlesi malaria vectors should be investigated in greater depth [216–221]. Next, landscape and urban planning should be integrated into the vector control program. A ‘buffer zone’ free of primates (humans and simians) and breeding ground for knowlesi malaria vectors should be created between human dwellings (or areas with human activities) and natural forests. Different species of anopheline mosquitoes require different breeding ground conditions [212, 222]. Hence, landscapes can be shaped to create an environment that is inconducive for the vectors to breed. Information such as the flight performance and flight distance of the vectors should be taken into consideration when designing the ‘buffer zone’ [212, 223]. For instance, *Anopheles balabacensis*, one of the established vectors for knowlesi malaria, was shown to have relatively weak dispersal capacity with maximal flight distance of 475 m [224]. Hence, in areas where *An. balabacensis* serves as vector for *P. knowlesi*, the size of the ‘buffer zone’ between human activity area and monkey habitats should be at least 1 km to increase the success of breaking the knowlesi malaria transmission circuit from monkeys to humans via mosquitoes. Physical barriers such as solar-powered electric fence (with electric current adhered to safety and ethical guidelines) can be built between the forest (monkey habitat) and ‘buffer zone’ to hamper monkey intrusion. In addition, these ‘buffer zones’ should not be open to the public. Nevertheless, they can be designed to serve multiple key purposes, such as flood control system, water reservoir, solar panel field, and aquaculture sites.

Cooperation and coordination between policy makers, law enforcement officers and public members are crucial to halt the transmission of zoonotic malaria. Local socioeconomic activities should be arranged accordingly to avoid outdoor activities during the feeding period of vectors. At the same time, public education plays an important role. High awareness about this zoonotic infection among the public will give rise to high compliance to measures against knowlesi malaria transmission. For example, public members, especially those involved in tourism, forest resource collecting and logging sector should be educated to plan their activities in parallel with the knowlesi malaria control programme. Farmers that practice subsistence cropping at the fringe of forests should be encouraged to adopt the much more productive farming techniques at relocated farm lands further away from the forests. Certain agricultural practices that employ simian primates such as the coconut harvesting should be replaced with simian-free alternatives. Besides, community relocation from forested areas to non-forested places equipped with better building design that reduces mosquito invasion and better layout of healthcare and sanitary infrastructure should be implemented proactively. All socioeconomic activities should be reviewed and approved by relevant authorities prior to implementation. The itinerary of the activities should minimize, if not completely avoid the risk of being bitten by the vectors. Furthermore, warnings regarding knowlesi malaria transmission and measures to prevent knowlesi malaria transmission should be stated clearly when promoting tourism in knowlesi malaria endemic areas. Foreigners should be informed clearly about this zoonosis before and upon arrival at the destination. Tourists should clearly report their travel history if they fall sick after returning to their countries of residence from the knowlesi malaria endemic region. A health alert card about knowlesi malaria may be given to travellers arriving at knowlesi malaria endemic areas. This may reduce the chance of overlooking *P. knowlesi* infection by healthcare workers in the travellers’ countries of residence if they came down with the infection after travel.

## Conclusions

The emergence of knowlesi malaria has definitely changed the dynamics of how we manage and control malaria towards a complete eradication from human population. The exponential increase of *P. knowlesi* research over the past few years has contributed to our greater understanding on the pathobiology, genomics and evolutionary biology of this parasite, at the same time improved our ability to detect this parasitic infection. The knowledge gap in several aspects about *P. knowlesi* that deserve more research attention in future has been elaborated. Knowlesi malaria further complicates the malaria eradication program. However, with tailor-made strategies, the transmission of knowlesi malaria in humans may be blocked without severely compromising the welfare of the simian natural hosts, wildlife biodiversity and economic development of knowlesi malaria endemic region.

## Data Availability

Not applicable.
