# Correction: *Plasmodium knowlesi*: the game changer for malaria eradication

**DOI:** 10.1186/s12936-023-04732-x

**Published:** 2023-10-19

**Authors:** Wenn-Chyau Lee, Fei Wen Cheong, Amirah Amir, Meng Yee Lai, Jia Hui Tan, Wei Kit Phang, Shahhaziq Shahari, Yee-Ling Lau

**Affiliations:** Department of Parasitology, Faculty of Medicine, Universiti Malaya, Kuala Lumpur, Malaysia

**Correction: Malaria Journal (2022) 21:140** 10.1186/s12936-022-04131-8

Following publication of the original article [1], it was brought to the journal's attention that the Funding declaration was incomplete. The declaration has since been corrected to the following: SS was supported by the Long Term Research Grant Scheme (LRGS/1/2018/UM/01/1/2) of the Ministry of Higher Education, Malaysia. YLL was supported by the Long Term Research Grant Scheme (LRGS/1/2018/UM/01/1/4) of the Ministry of Higher Education, Malaysia.

The authors thank you for reading this erratum and apologize for any inconvenience caused.
